# Profile of reintubated patients submitted to daily weaning screen and spontaneous breathing trial in a general ICU

**DOI:** 10.1186/cc12658

**Published:** 2013-06-19

**Authors:** NR Machado, CSM Silva, C Taniguchi, E Giovanetti, PA Pellegrini, D Carineli-Cazati, GFJ Matos, RC Eid, KT Timenetsky

**Affiliations:** 1 Hospital Israelita Albert Einstein, Morumbi, Sao Paulo, SP, Brazil

## Introduction

Mechanical ventilation (MV) weaning is the transition of artificial ventilation to spontaneous breathing of patients intubated for more than 24 hours. Reintubation may occur, even if the weaning process has been well conducted, in 13 to 19% of the extubated patients. Daily weaning screen and spontaneous breathing trial are widely used to evaluate patients ready to be weaned, although a reintubation risk may occur [1]. The objective of this study was to verify the profile of patients that failed the weaning process and needed to be reintubated.

## Methods

Mechanically ventilated patients submitted to our institutional MV weaning protocol from January to July 2012, who were extubated and failed extubation within a 48-hour period, were included in the study. The weaning protocol consisted of daily weaning screen and spontaneous breathing trial. Demographic data, MV time, ICU and hospital length of stay, causes of reintubation, and mortality rate were collected during the study period.

## Results

Two hundred patients were included, and 29 (14%) were reintubated. Of the reintubated patients, 59% were male, with a median age of 69 years (range of 24 to 94), mean Simplified Acute Physiology Score (SAPS 3) of 60 ± 11, mean MV time of 9 days ± 5, median ICU stay of 14 days (range of 5 to 30), and 46 days of hospital stay. Causes of reintubation were acute respiratory failure (38%), low level of consciousness associated with lack of airway protection (27%), and hemodynamic instability (14%). ICU discharge occurred in 70% of the patients, and 31% were tracheostomized due to dysphagia, low level of consciousness, or lack of airway protection. The ICU mortality rate was 30%. Only one tracheostomized patient died. Patients with ages ranging from 86 to 88 years had a higher incidence of low consciousness level. Patients that did not use noninvasive ventilation (NIV) after extubation were reintubated earlier than others (median of 20 hours, *P *<0.02 and *r *= -0.551), although there was no correlation with the use of NIV with mortality or MV time.

## Conclusion

The use of daily screening and spontaneous breathing trial is associated with a low reintubation rate. Acute respiratory failure and a low level of consciousness were the most common causes of reintubation, and most patients were discharged from the ICU. NIV may prevent the need for reintubation. Patients with no perspective of short-term improvement in level of consciousness may be considered for a tracheostomy.
